# Levels of Social Sharing and Clinical Implications for Severe Social Withdrawal in Patients with Personality Disorders

**DOI:** 10.3389/fpsyt.2017.00263

**Published:** 2017-12-04

**Authors:** Livia Colle, Giovanni Pellecchia, Fabio Moroni, Antonino Carcione, Giuseppe Nicolò, Antonio Semerari, Michele Procacci

**Affiliations:** ^1^Department of Psychology, University of Turin, Turin, Italy; ^2^Third Centre of Cognitive Psychotherapy, Rome, Italy

**Keywords:** social sharing, reciprocity, intimacy, avoidant personality disorder, mindreading, metacognition, social withdrawal

## Abstract

Social sharing capacities have attracted attention from a number of fields of social cognition and have been variously defined and analyzed in numerous studies. Social sharing consists in the subjective awareness that aspects of the self’s experience are held in common with other individuals. The definition of social sharing must take a variety of elements into consideration: the motivational element, the contents of the social sharing experience, the emotional responses it evokes, the behavioral outcomes, and finally, the circumstances and the skills which enable social sharing. The primary objective of this study is to explore some of the diverse forms of human social sharing and to classify them according to levels of complexity. We identify four different types of social sharing, categorized according to the nature of the content being shared and the complexity of the mindreading skills required. The second objective of this study is to consider possible applications of this graded model of social sharing experience in clinical settings. Specifically, this model may support the development of graded, focused clinical interventions for patients with personality disorders characterized by severe social withdrawal.

## Introduction

A fundamental characteristic of human sociality is that humans appear to have a specific aptitude for social sharing, cooperation, and reciprocity. Human social sharing skills have attracted interest from a wide range of disciplines: from philosophy, the social sciences and anthropology to psychology and, more recently, the neurosciences. Depending upon the field of research involved, the concept of social sharing has assumed very different connotations, and it has been studied from various perspectives and using diverse methodologies.

In order to arrive at a comprehensive definition of the social sharing concept, keeping in mind these diverse areas of research, we must take into account the various constituent elements of this kind of social interaction: the motivational element, or the impulse which induces human beings in early ontogeny to engage in it; the contents of social sharing, or the variety of mental states that human beings share with each other in social interactions; the subjective experience and the emotional responses it evokes; the behavioral outcomes of social sharing; and, finally, the mental functions or cognitive components which underpin this specific form of social interaction.

With regard to motivational aspects, most researchers agree that social sharing is a primary objective in itself (and not secondary to other interpersonal objectives), that it is innate and that it emerges at a very early stage of development. In early infancy, human beings already appear to possess an intrinsic motivation to initiate social exchanges and to engage in reciprocal interactions (1–3). Studies of infants have shown that 2-month-old infants display various emotional reactions to attention directed toward the self as well as to intra- and interpersonal coordination during face-to-face communication (4, 5). At an early age, young children also appear predisposed to share interests and goals with others (6, 7). Joint attention is considered a very early signal of the human predisposition for social sharing. At 9–12 months, infants are already able to coordinate their visual attention with others and are able to produce a triadic gesture involving not only the child and an adult but additionally an external event or entity. Specifically, in proto-declarative pointing, the infant uses pointing to an object as a tool for capturing adult attention and for sharing with the adult their own interest in an object or event (8, 9). Fourteen- to eighteen-month-old human infants do understand shared intentions and they respond accordingly (7, 10, 11). During their second year of life, infants extend their repertoire of social sharing behaviors: they engage in pro-social and cooperative behaviors. Toddlers help others in many ways, comforting those in distress or providing instrumental help. For example, they can help adults by pointing to provide needed information or by fetching out of reach objects (12–16). At the same age, their sharing behaviors also increase: toddlers offer toys to the adults and they point with the purpose of sharing interest with others (17). 18-month-old children demonstrate that they are able to collaborate with adults in joint activities, having a joint goal in mind. The children, for example, actively attempted to reengage the adult communicatively if adult unexpectedly interrupted the joint action and they participated enthusiastically in these social games, in which there was no material reward (12). By 3 years of age, there is evidence that children are beginning also to feel some of the commitments and obligation inherent in joint action themselves, as when they excuse themselves when they have to leave a joint action (18).

The enormous development of social media in recent years could be viewed as a further indirect manifestation of this innate and spontaneous human motivation to engage in social sharing. Users can now communicate their experiences with potentially unlimited numbers of people. In 2016, there were 2.34 billion social network users worldwide [see Ref. (19)].

Although social sharing is considered an innate and universal human need (16), in some clinical conditions, the absence of this desire predominates. This is the case with Schizoid Personality Disorder (20, 21) and also in the autistic spectrum disorders. Recent interpretations of the social difficulties of patients with high functioning autism suggest that their main difficulty is not primarily an impaired understanding of other people’s mental states, but rather an absence of motivation to engage in social sharing. In consequence, they are less sensitive to and less aware of the interpersonal information which is relevant for social bonding (22, 23).

Human social sharing can also be defined in terms of the mental state, the content, which is shared between the agents. Engaging in joint actions in order to achieve a common objective can be considered an example of social sharing. Even in such simple situations, for joint action to take place, the two agents must share the same intention and the same goal. Collective intentionality has been defined as the ability that allows human beings to develop joint intentions and joint commitments in cooperative actions (16). In one frequently cited example given by Gilbert (24), human joint intention is explained through the comparative analysis of two superficially similar actions. The first is a simple cooperative act, illustrated by the idea of going for a walk with a friend. The second is a random act, which to an external observer could appear indistinguishable from the first: two strangers are walking down a road side by side. The difference between these two similar actions becomes immediately apparent when we imagine what would ensue if one agent should suddenly walk off in another direction. If the two agents were *not* out walking together, this sudden deviation would have no relevance and would provoke no consequences. However, if the person veering off were engaged in a joint action, based on a pre-existing joint intention, this move would affect the behavior of the other and would most probably provoke surprise or protest at the violation of a joint plan of action (24, 25). Recent neuropsychological studies have produced substantial data on the neural mechanisms involved in dyadic, interactive, and mutual actions (26–32).

However, as Schweikard and Schmid (33) have emphasized, collective intentionality comes in a variety of different modes, which include shared intention, joint attention, shared belief, and collective emotion. For example, the precocious human capacity for joint attention can be seen as the foundation for another social sharing content: mutual knowledge or common ground (34). Common ground can be defined as the body of past shared experience held in common by the agents in a communicative interaction. This constitutes a shared frame of reference, an implicit and extended context that defines the ultimate meaning of the interaction. Common ground is therefore a crucial component of human communication, and it is considered a core feature of social relationships (16, 35). Child development studies have shown that once children have acquired the capacity for joint attention, at between 1 and 2 years of age, they begin to accumulate shared knowledge derived from their interactions with others and to make use of this shared knowledge in ways that are specific to the persons they are relating to Ref. (36).

Social sharing can also involve the emotional domain; in fact, many researchers use the term social sharing to refer exclusively to the social sharing of emotion (37). Affect sharing can involve implicit processes, as in the affective attunement during early mother–infant interactions (38), but it can also refer to the conscious sharing of emotional states, such as when we realize that we share the same enthusiasm for a work of art with another person or the same disappointment when our football team loses the game. Sharing of emotion requires high levels of sensitivity and responsiveness to the emotions of the other and has sometimes, for this reason, been considered as equivalent to or synonymous with the ability to empathize. However, social sharing is by no means restricted to states of emotion and encompasses a wide range of different mental contents. In other words, every type of mental experience can become an object of social sharing.

In some cases, the sense of familiarity arising out of social sharing situations can lead to a sense of intimacy or of bonding; Sullivan was the first to underline the connections between social sharing and this sense of intimacy. In his view, both the capacity for constructing close personal and social bonds with friends and romantic partners and the need for intimacy emerge between childhood and adolescence, when one’s closest peer relationships are likely to be with same-gender friends (39). Sullivan described intimacy as a collaboration in which both partners reveal themselves and seek and express validation of each other’s attributes and world views. Since close friends generally resemble each other, collaboration on establishing shared understandings and a sense of mutual esteem and emotional security is relatively straightforward. Other researchers have shown that between childhood and adolescence, children’s descriptions of friendship begin to emphasize this aspect of sharing intimate thoughts and feelings (40–42).

The mental states shared by the agents during social interactions can vary widely, ranging from the sharing of simple and immediate goals, to the common ground of shared knowledge, the awareness of a shared emotional experience, or the intimate knowledge shared with a partner or a family member. The hypothesis we will discuss here proposes that experiences of social sharing can be classified at different levels of complexity according to the type of mental content shared by the agents.

Social sharing can also be described in terms of the consequences it produces. One important consequence concerns emotional effects, or the subjective perceptions produced in the agents involved. Dimaggio et al. (43) discuss how people perceive different shared mental contents such as values, beliefs, emotional experiences, and interests, and they suggest that such perceptions form the basis for people’s sense of belonging to social groups. In Tomasello’s anthropological interpretation (2), social sharing with group members is the basis for affiliation processes. Along the same lines, recent neuropsychological data have shown that our personal experience of specific interactions conducted with others can influence the modalities of subsequent interactions and even the neural systems activated during those later social interactions (32). According to Procacci (44), enjoyment and pleasure in interaction are generally associated with social sharing, while withdrawal is linked to embarrassment and, in some clinical conditions such as avoidant personality disorder (AvPD), to shame, threat and severe isolation. The behavioral outcomes of social sharing, however, extend beyond the emotional domain. As research into collective intentionality has shown, the experience of social sharing facilitates cooperation and reciprocal commitment. People engaged in a joint action automatically take up a specific mental attitude (*we mode*), assuming that they are expected to play their part and that their counterparts will also contribute to the joint enterprise. In other words, a collective expectation arises that joint obligations will be shared and will be mutually respected by all the actors involved. Joint aims, intentions, or shared ideas are what shape cooperative and normative behaviors, choices which are based on respect for the rules of reciprocal commitment.

Finally, identifying the mental abilities involved is fundamental for an understanding of the social sharing experience. Although many diverse abilities and neurobiological mechanisms are implicated in the different forms of social sharing, we have described, for the purposes of this study we consider one ability, mindreading, which appears to play an important role in many forms of social sharing.

In our hypothesis, the fundamental precondition for social sharing is that individuals must be capable of forming internal representations of their own minds and of the minds of others, either through implicit elaboration of social information (through mimicry, for instance, or through the interpretation of facial expressions), or by processing explicit information. *Vice versa*, where mindreading capacities are impaired, we can expect a failure of social sharing (44, 45). As we will discuss below in greater detail, there is clinical relevance in identifying the role of mindreading in different types of social sharing experience, since a number of mental disorders are associated with some degree of impairment of interpersonal relationships and with social withdrawal, in particular in the case of the personality disorders. Patients with personality disorders are significantly affected by interpersonal difficulties (20, 46). In particular, lack of experience of social interaction and intimacy, as well as impairments in mindreading, appear to play a significant role in mental disorders characterized by chronic social withdrawal, such as avoidant, paranoid, or schizoid personality disorder (44, 47–49). We will argue in this study that classification of social sharing at different levels, according to the complexity of the contents and the complexity of the mindreading competence required, can provide a framework for the design of graduated clinical interventions to enhance the social sharing skills of patients with AvPD.

Summing up, social sharing is typical of human interaction and emerges very early in life, evolving into increasingly complex forms throughout development (38). Social sharing refers to a specific interpersonal experience involving two (or more) human agents, each of whom is able to form internal representations of the mental states shared with the other agent/s. The contents held in mind and in common by two (or more) agents, can be highly diverse and can range from a shared motor plan of action to the communication of personal or intimate information. Social sharing is accompanied by positive emotions, such as mutual enjoyment, all of which can promote reciprocal commitment, cooperation, affection, and the sense of belonging. Certain preconditions must be met if social sharing is to take place: agents must be motivated to engage in social sharing and their mindreading skills must be fit to meet the specific demands of processing more or less complex mental contents involved in different types of social sharing.

In this study, we will first of all discuss the inter-relationships between social sharing and mindreading skills. We will argue that there is a reciprocal, bi-directional influence between the two. While mindreading competence is a necessary precondition for social sharing, it is also the case that social sharing experience modifies and enhances mindreading skills. Over time, a virtuous circle evolves with repeated and varied experiences of social sharing sharpening and upgrading people’s mindreading skills. We will present here a model of different type of social sharing experiences classified according to their complexity. The simplest levels of social sharing involve simple contents which, therefore also require a minimum of ability to attribute mental states to others (mindreading). The more complex levels of social sharing involve more complex states of mind during the interaction and make greater demands on mindreading competence.

In the second part of this paper, we will focus on some forms of psychopathology in which an inversion of the positively reciprocal relationship between social sharing and mindreading takes place, producing the vicious circle of social withdrawal and isolation. Additionally, we will show how a model of different levels of social sharing can inform the development of more focused, graduated clinical interventions for PD patients with severe social withdrawal.

## Mindreading and Four Levels of Social Sharing

As we showed above, there are various human interactions which can be considered as expressions of social sharing. The aim of this work is both to differentiate different types of social sharing and to classify them according to levels of complexity. In our hypothesis, the two main constituent elements involved are (1) the type of mental content shared by the agents, and (2) the level of mindreading skill which each agent must possess in order to understand and evaluate what is being reciprocally shared. In the preceding section, we showed that social interaction can encompass the sharing of diverse mental states: intentions, attention, knowledge, emotions. In the course of one interaction, it is possible to share one or more of these diverse mental states. We hypothesize that social sharing can be classified in terms of complexity of mental content.

To illustrate this point, we may imagine two very different social interactions based on an identical scenario. The scenario involves two individuals at a party, who are asked to move a heavy table together. In the first scenario, the two agents do not know each other, while in the second scenario they are old friends. We can imagine that in the first situation the two agents restrict their interaction to coordinating the table-moving operation and that their conversation is focused primarily on the task in hand. In the second scenario, we can imagine a very different style of communication. Two friends who have not seen each other for some time are asked to move a heavy table into the garden. Reminiscing about a removal they once worked on together, one starts joking with the other about his poor performance on the previous occasion. He keeps up a running commentary about the poor shape his friend is in, possibly even worse than the time before, while obstructing every maneuver, trying to trick him into making false moves and teasing him relentlessly. The other one joins in the fun, retaliating in kind.

Although the joint action in both scenarios is identical (moving a table together), the mental contents involved in each case are only partially similar. Each pair shares the same goal and reciprocal commitment (moving a table) and, with this aim in view, each agent monitors his own movements and modulates his own actions in coordination with those of his partner. The motor planning and the joint attention required are the same in each scenario. The outcome of this type of sharing is in each case a joint action regulated by cooperation.

However, in the second scenario, the social sharing involved encompasses additional mental states and operates at a far higher degree of complexity. Both agents share a memory and a frame of reference deriving from their past experience together, which constitutes an implicit body of knowledge that both agents share in and can draw on during the current interaction (common ground). In other words, the experiences lived through together in the past are being reactivated in interpersonal communication here and now, and there is a reciprocal awareness of this fact. The irony and the reciprocal teasing that characterize this interaction are based on reciprocal knowledge of past shared experience.

A comparison between these two scenarios makes plain how even a very brief and simple social interaction such moving a table together can comprise qualitatively very different experiences of social sharing and reciprocity, depending on the different types of mental states which constitute the interaction such as shared intention, shared memories, common knowledge, emotional attitude. We therefore hypothesize that the type of mental content being shared may be considered a crucial element for the classification of different experiences of social sharing.

If an individual is to take part in interpersonal communication based on social sharing, and if they are to experience, this as an enjoyable activity, they must be capable of representing to themselves the mental states involved in the interaction. As we have described above, mental states can vary greatly from interaction to interaction. We therefore consider mindreading ability as the second fundamental element of social sharing, and that social sharing interactions can also be classified according the complexity of the mindreading skills required in a given interaction.

Mindreading is the mental function that allows us to observe and reflect upon mental states, either our own (e.g., what do I feel about the other person or what do I want from them), or those of others engaged in interactions with us (e.g., what are his intentions, what sort of mood is she in, what does she think about a specific topic). Mindreading, mentalization, or metacognition is different terms which all refer to a general ability to understand and reflect on both our own mental states and other people’s (50–53). The way we use the term mindreading in this study relates back to a wider concept of mentalization as defined by Allen et al. (54), meaning “to keep the mind in mind.” Thus, mindreading refers to a complex of implicit and explicit operations centered around understanding mental states, one’s own and those of others; operations which are deployed by individuals in their everyday interactions with varying degrees of proficiency and success (51–53, 55). Mindreading is a mental ability which is considered fundamental for the gradual development of social competencies (38) from early infancy onward and into adulthood (56). Consistently with this view, the early mindreading impairments that accompany autistic spectrum disorders are seen as playing a significant role in the profound social difficulties which are characteristic of the autism spectrum.

If mindreading capacities are key to the development of social competence in general, we would also expect them to be involved in that specific modality of social interaction which is social sharing. We hypothesize that social sharing and mindreading can be viewed as tightly related and that a better understanding of this relationship may also serve various clinical purposes, as we discuss below.

If we take a long-term perspective, over a period of years, the interaction between experiences of social sharing and mindreading skills appears as a factor which may support or impede the development of satisfying and stable interpersonal relationships. Motivation for social sharing stimulates individuals to take an interest in other people’s mental states and to pay attention to them. Simple forms of such interest, together with attention to interpersonal signals, have been observed even in new-born infants, who appear to prefer human faces to other visual stimuli and to be more attentive to human faces than to other visual stimuli (57). This early interest in social signals puts infants on track to eventually codify the various implicit and automatic signals involved in understanding the minds of others (58, 59). In turn, from infancy onward, mindreading skills can facilitate and enhance social sharing experience. Numerous studies have supported the hypothesis that mindreading capacity is fundamental for creating, regulating and maintaining positive interpersonal relationships (60, 61). The literature on child development highlights the importance of the quality of early interpersonal relationships for the development of mindreading. Attachment theory, for example, proposes that early child/caregiver relationships affect children’s emotional functioning at all levels, from emotional awareness to emotion regulation strategies [e.g., see Ref. (62, 63)]. Similarly, some studies have found that the mentalizing capacity of caregivers, or the extent to which they are able to comprehend the child’s internal states and to reflect them back accurately to the child, represents a strong predictor of adult mindreading ability (64, 65). Halberstadt et al. (66) found that children who show high ability to understand emotional cues in their social environments generally develop superior social skills and form healthy interpersonal relationships. Moreover, a positive relationship has been found between children’s emotional understanding and their pro-social behavior and also their acceptance and popularity with peers (67–69). Conversely, poor understanding of emotions and failure to represent interactions in terms of mental states has been associated with a wide range of psychiatric conditions (70). Reciprocal inter-relations between mindreading capacity, social sharing, and psychopathology will be discussed in the section below.

We use mindreading capacity not only to interpret the mental states of others but also to access our own mental states. This is an aspect which has been less studied by developmental psychologists, but it does seem to be the case that the ability to understand one’s own internal mental states also plays a role in fostering or in inhibiting social sharing experience. Access to our own mental content enables us to identify what we are feeling, thinking, or wishing for, and to establish connections between this content and the person we are engaging with. Awareness and recollection of a specific emotional memory may motivate us to seek out further opportunities for social interaction with that particular person, or to seek similar levels of communication in other interpersonal relationships (affiliative or other close relationships). People who are aware that sharing an objective, a memory or an emotion can enhance their sense of subjective well-being are more highly motivated to seek further opportunities for similar social sharing experiences. The experience of social sharing also promotes affiliative and cooperative behaviors among members of the group, and these behaviors may in turn promote new social sharing experiences. This bi-directional interplay between mindreading skills and social sharing originates in the infant’s early experience with caregivers (65, 71), and continues to develop during childhood and adolescence. Repeated experiences of social sharing promote the development of more sophisticated mindreading skills, which in turn facilitate more meaningful experiences of social sharing. Between peers in adolescence, for example, sharing private or personal mental states can provide opportunities to compare one’s own emotionally significant experiences with the experiences of others (40). Knowing how other people feel before an exam, for instance, makes it possible to differentiate between common emotional responses and those which are more personal and specific to individuals. These interpersonal communications are useful not only in the sense that they convey information about other minds but also because they enable better understanding of one’s own mind. The interplay of mindreading and experience of social sharing may create virtuous as well as vicious circles. In psychopathological disorders, such as in patients with personality disorders, there is a bi-directional relationship between impaired mindreading and lack of social sharing experience, each element aggravating the other.

This hypothesis of an interlocking relationship between mindreading and social sharing is of interest, in our view, not only because of these long-term effects on individuals but also because it allows us to understand more clearly what underlies the distinctiveness of different types of social sharing interactions. As we illustrated through the two table-moving narratives, the joint action in each case required the agents to form a mental representation of a “we” mode, but the shared mental content in each case was widely divergent. There was a significant difference both in the content shared and in the complexity of the mental processing (mindreading) required of the two agents. As in the first example with the two strangers, there is not always a need to reflect in depth on mental states of others. There are many spontaneous social interactions in which, as in the first scenario of moving a table, a minimum of mindreading capacity is required. However, when relationships are prolonged over time, as happens when people spend significant periods of time together pursuing similar personal objectives, the content of social sharing can gradually become more complex. Sharing similar experiences and emotions may develop further into reciprocal knowledge, or common ground, and reciprocal empathy. Prolonged social interaction can in this way create a dynamic which allows individuals to achieve highly diversified and complex levels of mutual understanding. At its most complex level, this is the kind of understanding of other minds which can be experienced in close friendships or couple relationships.

We propose here four different levels of social sharing, based on the contents shared during the interactions and the complexity of mindreading skills required:

(1) First level: *shared short-term goals*. In these cases, social sharing is connected with a precise short-term objective and may involve an action, a communicative exchange, or joint attention focused on the same event. In such interactions, the reciprocal mindreading required for successful accomplishment of the action is minimal. To paraphrase a famous example from Fodor (72), all you need to do if you want to meet somebody somewhere is to imagine that the other person intends to meet you (shares our objective) and that you both intend to meet each other on a certain day, at a certain place and time. When people share a short-term objective, such as the two strangers in our first example who are moving a table together at a party, the mindreading activity involved can be described as joint attention focused on the same event and as a reciprocal understanding of the intentions underlying the actions of the other agent.
(2) Second level: *common ground*. Actions or experiences that individuals take part in together create opportunities for joint attention and for the constitution of a body of shared knowledge, a shared frame of reference, which is termed common ground. Our second illustration with the two friends moving a table shows how common ground can function as a resource, which can be drawn upon to create mental representations of reciprocally shared and known contents. In this sense, sharing knowledge can also be a mindreading operation, since it requires each agent to form a mental representation of the other agent’s previous knowledge of or beliefs concerning a specific past experience. The common ground of the two friends could be described in the following terms: *I know that you know X (or that you believe or remember X). You also know that I know this too and that I have clear memories of X. This allows us both to allude to X in ways which are not necessarily and immediately comprehensible or accessible to others*. In other words, interactions like the one illustrated here presuppose that each agent has the resources to engage in a complex activity of attribution of mental states to the other. Mindreading in this example operates at a more complex level than in the first story, where joint attention is called for and an understanding of the other agent’s intentions as they relate to the physical action of moving the table. In the second story, the two friends engaged in moving the table use the same skills, but additionally they are constantly referring back to a range of shared memories of past activities. During the interaction, each agent is therefore actively creating a rich representation of what the other knows and remembers of these previously shared experiences, and of how they relate to the current activity. Allusions to their common ground are key to the quality of this complex and enjoyable interaction.
(3) Third level: *emotional social sharing*. People sharing emotions are not only experiencing the same event or the same activity (and the reciprocal knowledge of that event or activity). They are also experiencing the same or similar emotional attitude, as friends do when they laugh at the same joke or shed tears at the same movie. In such situations, the contents being shared are emotions, or are related to emotions and to emotional reciprocity. Interactions at this level require the use of common ground, but with the additional element of reciprocal communication of emotion; these interactions may assume connotations of greater personal significance. This could be stated as: *I know that you feel as I do about X, or that your feelings are similar. Over and above that, I know that you know that I share your feelings*. People who have shared emotions with each other are often interested in creating further opportunities for interaction. This increases the probability that more time will be spent together, which in turn may create favorable conditions for emotional bonding and stable relationships.
(4) The fourth level: *familiarity and intimacy*. Close friendships and intimate, stable relationships are characterized by a complex and highly articulated level of social sharing and bonding. Typically, such bonding is associated with having spent substantial periods of time together and with an accretion of common ground and of shared emotional experience which allows each partner to construct a rich and coherent body of knowledge about the other. The salient point here is the development of deep reciprocal knowledge, rather than the nature of the relationship itself. For example, in the context of a sexual relationship, reciprocal sharing of personal and intimate contents may take place. However, such sharing is not a necessary component of a sexual relationship.

Close friendships and romantic relationships are associated with complex, meaningful reciprocal knowledge, and complex mindreading. Each actor can draw upon rich resources of information about their partner. This enables them to form highly articulated representations of the mind of the other, such as nuanced descriptions of their partner’s personality or intuitive predictions of their probable responses to specific situations. Reciprocal knowledge at this level allows us to modulate and maintain stable, meaningful relationships with the people who are closest to us.

Intimacy can be experienced both in small groups of people and in dual relationships. In a small group of friends, for example, it is possible that A, B, and C are all in possession of a certain body of shared knowledge, that they all feel the same way about certain past experiences they shared, and that they have developed deep reciprocal knowledge of each other over time, through changing circumstances and as they grow older together. A, B, and C are all reciprocally aware of this shared inheritance of group experience, and on this basis, they may develop a sense of intimacy which is specific to this particular group. It follows that each member of the group will respond to social occasions together with a sense of intimacy and sharing which is very different from that experienced in social situations with others who they know less well. However, since these are intimate and private relationships, it is also possible to imagine that each member of the group may develop a dual relationship with each other member, and that such relationships may have some particular quality which is specific to that relationship alone. The reciprocal social sharing and reciprocal knowledge between A and B, for example, may not be the same as between A and C, or B and C.

We suggest that looking at human social sharing in terms of different levels of complexity as we do in this model offers a number of advantages in the clinical context. As we will discuss in the next section, model provides us with a structured framework for clinical intervention in cases of personality pathologies where the improvement or enhancement of social interaction is a priority.

## Social Sharing and Personality Disorders

In the first section of this paper, we propose that a relationship of interdependence exists between social sharing and mindreading and that social sharing can be described in terms of levels of complexity which are determined by the complexity of the mindreading operations involved in a given interaction. In the following section, we will attempt to illustrate the relevance of this dynamic of interdependence for the clinical context. In our view, the concept of a dynamic relationship between social sharing and mindreading is of clinical interest for two different reasons: first, because linking mindreading and social sharing can extend our understanding of specific forms of social withdrawal in personality disorders and, second, because differentiating and describing social sharing in terms of levels of complexity can support the development of specific interventions for the treatment of social withdrawal in personality disorders.

Social withdrawal is arguably a trans-diagnostic core feature of psychopathology (73). Difficulties with social interaction and the absence of stable networks of social relations feature in the clinical descriptions of many conditions and disorders, from the autism spectrum disorders to personality disorders and social phobia (SP).

There are three main factors, differently combined, which are associated with social withdrawal symptoms: (1) low motivation, (2) anxiety specific to social settings, and (3) inability to construct satisfying interpersonal relationships.

Motivation, as we mentioned earlier, is a precondition for social sharing. This motivation is already present in early infancy, and it is fundamental for the promotion of social interactions, for the acquisition of mindreading abilities and for the practice of perspective-taking. However, the absence of motivation to engage in close interpersonal relationships is a significant factor in some psychopathologies; and in some neurobiological conditions, such as Schizoid Personality Disorder or high functioning autism (20, 21, 74), this motivation to engage in close interpersonal relationships is typically absent. Social withdrawal is also present in many disorders where patients are actively motivated and interested in social sharing but find it hard to put into practice. Particularly patients with SP and AvPD appear to be motivated toward social sharing, but their efforts and aspirations to construct secure, meaningful, interpersonal relationships either end in failure or are associated with anxieties, embarrassment, and constant exertion (75, 76). SP and AvPD have frequently been considered as two very similar disorders, each characterized by social anxieties and differing only in intensity, with SP as the mildest form (77). However, recent studies have highlighted some qualitative differences between AvPD and SP, which appear to be related specifically to the capacity for creating opportunities for intimacy and social sharing with others (78–80). Millon (81) suggested that patients with AvPD fear *relationships* with other people, whereas patients with SP fear *social situations*. In other words, fear of social situations is related to specific social contexts (parties, large groups, meetings), which are experienced with excessive anxiety and shame. Fear of relationships, on the other hand, is linked with more basic interpersonal dysfunction involving avoidance of intimacy and chronic feelings of shame and inadequacy (78, 82, 83). Dimaggio et al. (79, 80) defined this structural interpersonal dysfunction in APD as a pervasive sense of not belonging. This sense of not belonging is likely to inhibit the development of social interactions based on affiliation and cooperation.

In the context of this current debate on the differences between social withdrawal in SP disorders and in AvPD, it may be useful to consider the way social sharing and mindreading interact. Many patients with social anxiety disorder who present avoidant behaviors in certain situations are not always reluctant to engage in interactions, neither are they incompetent at relating to others under all circumstances. Their fears of being judged by others are restricted to specific contexts of interaction. In other contexts, they may be capable of constructing close personal relationships and of enjoying moments of social sharing. Patients with AvPD are similarly anxious about social situations but, unlike the SP patients, and regardless of the setting or the circumstances, they are unable to enjoy or find satisfaction in social relationships and unable to construct meaningful interpersonal relationships. Summing up, patients with AvPD can be considered as experiencing “severe” social withdrawal for the following reasons: they experience pervasive fear of all types of interpersonal relationship, constant avoidance of intimacy, a chronic perception of not belonging and non-affiliation, and a diffuse sense of inadequacy which is not limited to specific social situations, as it is in the case of patients with SP. Millon has a concise description for the difference between AvPD patients and SP patients: patients with AvPD fear relationships rather than social situations (81).

Our hypothesis is that in the case of AvPD, anxiety, and impaired social sharing skills interact with and potentiate each other, producing a dramatic vicious circle in which patients are negatively affected both by isolation and by their experiences of interaction with others.

We have described social sharing as a positive interaction which presupposes both positive interpersonal relationships and mindreading ability. Mindreading functions as the core skill which enables the development of social sharing at various levels of complexity. Social sharing enhances interpersonal relationships. In turn, and over time, positive interpersonal relationships provide opportunities to improve and enhance mindreading skills.

In patients with AvPD, this process appears to manifest itself in reverse, as a vicious circle, in which poor mindreading and lack of social sharing experience sustain each other in turn. This hypothesis can be supported by two types of empirical evidence: (a) data which show that patients with AvPD have difficulties with mindreading and (b) data which show that the developmental history of these patients reveals a lack of interpersonal relationships.

Several authors are in agreement that the interpersonal difficulties typical for Personality Disorders are tightly linked to patients’ poor understanding of their own mental states and those of others (45, 80, 84). Mindreading difficulties in PD patients have also been described in terms of differentiated profiles of impairment: individual patients may present specific profiles of dysfunction, with some mentalization skills more impaired than others (53).

For example, the mindreading impairments observed in AvPD patients appear to lie mainly in the areas of emotional awareness and alexithymia (85, 86). Patients with AvPD not only lack awareness of negative emotions but also have low awareness of pleasurable emotions and enjoyment (82, 87, 88). Analysis of session transcripts of patients with PDs indicates that those with AvPD and Narcissistic PD showed the greatest difficulty in identifying emotions and their causes (79). These findings are corroborated by recent data which show that specific difficulty in identifying and reporting inner states (such as emotions, thoughts, or intentions) is strongly correlated with a “withdrawal” personality style (45). Similar findings are reported by Eikenaes and colleagues. Comparing AvPD with SP patients, they found that those with AvPD have a more vulnerable sense of self and are less self-reflective than patients with SP (82). AvPD patients scored as healthy control group in identifying different emotional expressions, except for fear (89). Another comparative study of AvPD patients, SP patients and patients with other personality disorders, found that the AvPD patients were less able to identify mental states in themselves and in others (49, 90).

Experimental findings have shown that there is an association between poor mindreading skills and the interpersonal relationship difficulties observed in AvPD. Fear of being judged, social anxiety, the sense of shame and avoidance are all emotions and social behaviors which appear to be strongly linked to deficits in social sharing, reciprocal knowledge, and intimacy. In their social interactions, these patients rarely perceive other agents as peers, as basically well-meaning and supportive individuals who may even resemble them in some ways in their aspirations to friendship, shared experiences, and meaningful relationships (91). The developmental history of patients with AvPD is characterized by low frequency of interpersonal relationships among peers, often limited to standard social situations such as school, and by difficult family relationships. In other words, there is a marked lack of intimate relationships and enjoyable social relationships in the lives of these patients. They have often experienced ostracism and social isolation during childhood and adolescence (48, 82). Such experiences of chronic social rejection and exclusion increase the likelihood that social interaction will be interpreted as threatening and they are strongly associated with social anxiety and isolation (92). In short, patients with PD characterized by severe social withdrawal typically present with the following profile: (a) poor mindreading ability regarding both self and others; (b) a developmental history characterized by few opportunities to socialize and limited time spent with others; and (c) few individuals available for enjoyable and satisfying interpersonal communication and not infrequently nobody available at all.

The social sharing model we described here can be used by clinicians to define the objectives of therapy with greater clarity. For example, patients with AvPD frequently report dissatisfaction at their lack of social relationships. However, their mindreading deficits and their limited experience of functioning social relationships may lead them to imagine relationships built on rank and dominance, rather than around social sharing. Since the lives of these patients are typically conditioned by experiences of exclusion and humiliation, some may cultivate fantasies in which the experience of submission and victimization is reversed, imagining themselves in dominant and abusing roles (44). In such cases, the therapeutic objectives must therefore include learning to distinguish clearly between social interactions based on rank and dominance and the more functional and enjoyable interactions which are based on reciprocity and social sharing.

This social sharing model may also serve as a basis or framework for the treatment of impaired social skills. As we discussed above, one important difference between patients with AvPD and patients with SP is related to the presence or absence of mindreading skills, which enable people to construct stable, satisfying relationships (49). This difference has a significant impact on treatment decisions. In the case of patients with AvPD, treatment will produce results only if accompanied by gradual acquisition of the social sharing and mindreading skills which will enable functioning interpersonal relationships. With such clinical applications in mind, the social sharing model presented here has the advantage of differentiating acts of social sharing in terms of levels of complexity. The model provides a rationale for the step by step, incremental learning of social sharing skills in a group therapy setting with a specific focus on social skills training. In parallel, patients attend individual therapy sessions. The group therapy uses an explicit psycho-educational approach to teach or to raise conscious awareness of the mindreading skills and the basics of social sharing which these patients lack. Importantly, the group social skills training also provides a safe space for real interactions, a therapeutic setting where theoretical awareness can be translated into practice with the guidance and monitoring of the therapists. In other words, the group therapy has a dual objective: teaching patients explicitly to reflect not only on social sharing and on thinking about thinking but also providing a practical, real-life opportunity to experience social sharing and mindreading directly.

This social skills training group is made up of patients with AvPD and associated difficulties. Two therapists work together to introduce the different elements and stages of social sharing. Patients are guided gradually toward direct experience of various levels of sharing through role play in the group, class discussions, and exposure exercises. The program is composed of two modules, one with a focus on mindreading skills and the other focused on the different levels of social sharing. The mindreading module addresses the acquisition of different mindreading skills. This module includes various practice exercises which aim to enhance patients’ awareness of their own mental processes and emotions and to improve their understanding of the mental states of others. Through group discussions, the module also aims to improve patients’ ability to assume perspectives other than their own (perspective-taking, or decentering), and to evaluate the mental states of others as existing independently and not necessarily connected to our own. The social sharing module is based on the framework of levels of complexity described above. As we discussed in the introduction, social sharing is the basis of both cooperative behaviors and the individual’s sense of belonging to the group. Both are generally severely impaired or absent in patients with AvPD. For this reason, social sharing experience is initially modeled through very simple tasks, such as sharing an immediate goal in the here and now, e.g., by forming teams for a game. These activities provide a first opportunity for brief, focused communication with others on specific topics (Short-Term Goal). Over time and through repeated moments of interaction, the members of the group begin to discover and to share different relational challenges, to recognize similarities and differences in each other and to share more explicitly a long-term goal: learning how to practice the essential skills that underpin effective social interaction (Long-Term Goal). These group sessions take place in a relaxed and good-humored atmosphere. Their purpose is not only to discuss and reflect on aspects of social sharing but also to enable concrete emotional experiences which are positive and enjoyable in themselves (Emotional Social Sharing). We consider that sharing enjoyable social experiences is a key component for the promotion of the sense of belonging and of cooperative behaviors. By the final stage of the group experience, the members know each other better and can begin to experiment with the personal and interpersonal effects of confiding in another person and sharing information of a more personal nature, taking turns as speaker and listener. At this stage, patients may begin to acquire an understanding of the more complex level of social sharing that encompasses friendship and intimacy (Familiarity and Intimacy). As they progress from simple to more complex social sharing experiences in the protected group setting, patients are also actively engaged in step by step improvement of their mindreading skills within the group. Until they have acquired an adequate understanding of the way other minds work, it may be not only difficult for such patients to establish close interpersonal relationships outside supportive settings but even risky, given their poor mindreading abilities and their unrealistic expectations of interpersonal relationships. Starting off at the simpler levels of social sharing allows patients to practice mindreading in a monitored, supportive setting and to regulate levels of social interaction according to their own growing capacity for mindreading. Once patients understand that social exchanges need not necessarily involve highly personal or sensitive material, they can be encouraged to engage in social and communicative interactions outside the group, with appropriate backup and reassurance. During the skills training, patients develop awareness of social sharing as an inherently enjoyable aspect of social interaction. They are also made explicitly aware of the fact that social sharing requires differentiated, flexible approaches and that there are many different ways of engaging in social sharing. Positive experiences in the group and enhanced awareness of mindreading skills may prompt patients to explore further options for social interaction and their own availability for such interactions outside the clinical setting.

We suggest that this incremental, structured approach to social skills training in group therapy settings may provide an efficacious method of treatment, allowing clinicians to address directly the interpersonal difficulties which characterize personality disorders. Further studies will be required to test the effectiveness of the proposed treatment model.

## Author Contributions

LC provided a fundamental contribution for creating the theoretical model proposed, taking together the results of different domain on social sharing. She was also responsible for drafting the initial version of the manuscript. She was responsible also of integrate every suggestion and comments proposed by co-authors. GP provided relevant contribution to design the clinical work and offer useful comment to improve the initial version of the manuscript. He was also responsible to ensure for the accuracy and integrity of every part of the work. FM helped to design the clinical work and offer useful comment to improve the initial version of the manuscript. He was also responsible to ensure for the accuracy and integrity of every part of the work. AC provided useful suggestions for the design of the clinical application of the model. AS offered relevant contributions to the brain storming for the conceptualization of the model and useful suggestions for the design of the clinical application of the model. He carefully revised the different versions of the manuscript until final approval. He was also responsible to ensure for the accuracy and integrity of every part of the work. GN provided useful suggestions for the design of the clinical application of the model. MP offered relevant contributions to the conceptualization of the theoretical model.

## Conflict of Interest Statement

The authors declare that the research was conducted in the absence of any commercial or financial relationships that could be construed as a potential conflict of interest.
